# Relay intercropped soybean promotes nodules development and nitrogen fixation by root exudates deposition

**DOI:** 10.3389/fpls.2024.1447447

**Published:** 2024-12-20

**Authors:** Ping Lin, Jin Wang, Ping Chen, Zhidan Fu, Kai Luo, Yiling Li, Tian Pu, Xiaochun Wang, Taiwen Yong, Wenyu Yang

**Affiliations:** College of Agronomy, Sichuan Agricultural University/Sichuan Engineering Research Center for Crop Strip Intercropping System/Key Laboratory of Crop Ecophysiology and Farming System in Southwest, Ministry of Agriculture, Chengdu, China

**Keywords:** maize-soybean relay strip intercropping, root exudates, flavonoids, nodule development, endophytic rhizobia

## Abstract

**Background:**

Legumes, in the initial event of symbiosis, secrete flavonoids into the rhizosphere to attract rhizobia. This study was conducted to investigate the relationship between crop root exudates and soybean nodule development under intercropping patterns.

**Method:**

A two years field experiments was carried out and combined with pot experiments to quantify the effects of planting mode, i.e., relay intercropping and monocropping, and genotypes, i.e., supernodulating NTS1007(NTS), Nandou-12(ND) and Guixia-3(GX) on root exudates, rhizobium community structure, nodule development and nitrogen fixation ability.

**Result:**

The result demonstrated that, maize–soybean relay strip intercropping not only promoted daidzein and genistein exudates of soybean root to soil but also reshaped the community structure and diversity of nodule endophytic rhizobia. Compared with monocropping, the nodule number significantly decreased in relay strip intercropping soybean, and NTS achieved 97% at soybean five trifoliolate stage. At soybean full bloom stage, despite the nodulation capacity of relay strip intercropping soybean was unrestored, the nodule number, nodule dry weight, nodule diameter and root dry weight were the highest in ND under relay strip intercropping. Compared with monocropping, the nodule average diameters of ND and GX in relay strip intercropping significantly increased 26.30% and 21.11%, respectively, the single nodule nitrogenase activity and *nifH* gene was increased up to the higher level of 3.16-fold and 1.96-fold, 70.8% and 107.6%, respectively. Combined with pot experiments, the nodule number of ND and NTS in maize root maize root exudates (RE) treatment increased with growth period, the GX reached its maximum at full bloom stage. And the nodule diameter of ND under RE treatment showed the best response. At R2 stage, compared with distilled water (DW) treatment, the nodule average diameter of ND and GX in RE treatment was significantly higher, and the *GmEXPB2* gene was significantly up-regulated 3.99-fold and 1.02-fold, respectively.

**Conclusion:**

In brief, the maize–soybean relay strip intercropping enhanced the soybean root exudates nodulation signaling molecules, meanwhile, maize root exudates caused increased nodule diameter, and enhanced nodule nitrogen fixation, but had little effect on supernodulation varieties.

## 1 Introduction

Root exudates are an important component of the organic matter returned by plants to the soil, altering the chemical composition of the soil surrounding the roots to influence the reshaping of the rhizosphere environment for soil microbial growth (Hu et al., 2018). Then, plants recruit specific rhizosphere microbial communities and advance growth and development in response to change in their growth stage or environmental stress (Huang et al., 2014; Backer et al., 2018). Isoflavones, which occur mostly in legume plants as functional components, mainly include daidzein, genistein, and glycitin (Aoki et al., 2000). In symbiosis, isoflavones, such as daidzein and genistein, are secreted into the rhizosphere and can promote the binding of roots and rhizobia in leguminous crops, where these compounds signal rhizobia to induce the rhizobia Nod gene, and then nodulation gene expression in root, such NIN, final form nodules on the roots (Weston and Mathesius, 2013; Kosslak et al., 1987). In legumes, the expression of gene for key enzyme, such as chalcone synthetase (CHS), is involved in flavonoid synthesis. The interference and silencing of *MtCHS* inhibited concentrations of root flavonoids and significantly reduced the nodulation in *Medicago truncatula* (Wasson et al., 2006). Isoflavone synthase (IFS) is a key enzyme that regulates the synthesis of the isoflavone, such as genistein and daidzein, by the redirection of flavonone intermediates, naringenin and liquiritigenin in the phenylpropanoid pathway (Sreevidya et al., 2006; Jung et al., 2000). However, soybean nodulation requires isoflavone synthase (*GmIFS*), which highlights the different needs of legume plants for isoflavones (Subramanian et al., 2006). In field cultivation, daidzein and genistein were found in soybean rhizosphere soil, and the ratio of daidzein to genistein was also higher in roots (Sugiyama et al., 2017). However, the capacity of crop roots to secrete flavonoids is also affected by many factors, such as climatic conditions, basic soil fertility and different planting patterns.

Soybean (Glycine max L.) is a crop that can provide nitrogen fixation for its growth and development, thus reducing the demand for nitrogen fertilizer (Qin et al., 2013), and it is also one of the main legume crops grown under the intercropping system. Through niche separation and complementarity, and the combination of high and low crops, the intercropping system realizes the full utilization of aboveground light resources (Raza et al., 2019; Liu et al., 2017). Not only that, tthe high morphological plasticity of crop roots promotes the niche complementarity of underground space in the intercropping system and improves the resource utilization efficiency, such as grass and legume intercropping (Zhang et al., 2022; Raza et al., 2023), for example, legumes intercropped with gramineaes. However, the severe competition for available resources (e. g., sunlight, water and nutrients) in the intercropping system directly affects the growth and development of soybean plants (Chen et al., 2017; Liu et al., 2017). To date, various field trials have been carried out on soybean intercropping, for example, in combination with maize, sugarcane or wheat (Nelson et al., 2011; Yang et al., 2013; Li et al., 2021). Interestingly, this systems can improve crop productivity by making efficient use of limited resources without affecting the environment (Li et al., 2021; Liu et al., 2018; Yong et al., 2018).

In the maize-soybean relay strip intercropping system, maize is usually sown two months earlier than soybean, therefore, the growth environment of soybean during the whole growth period is divided into two stages, namely, the shade period when maize and soybean coexist (mainly the vegetative growth period of soybean) and the natural sunshine period after maize harvest (the reproductive growth period of soybean) (Wu et al., 2021). It has been reported that maize shade significantly limited the development of leaf area in soybean plants, and decreased chlorophyll content and photosynthetic rate in the maize-soybean relay strip intercropping system (Fan et al., 2018; Wu et al., 2017), meanwhile soybean plants invest the biomass in stem elongation to adapt to the environment and improve light capture (Gong et al., 2015). Furthermore, soybean produced thinner and smaller leaves under intercropping conditions than the corresponding leaves under monocropping conditions (Fan et al., 2018; Wu et al., 2018). Studies suggest that the current supply of photoassimilates is affected by changing plant populations, shade or leaf area (Islam, 2014; Ahmad et al., 2015; Wu et al., 2016). Therefore, before and after maize harvest caused differences in photoassimilate supply in different growth environments of relay strip intercropping soybean. Intercropping promotes root growth and alters root distribution (Li et al., 2006). Maize–soybean intercropping systems increase soybean root growth plasticity, improve soil water use efficiency and phosphorus and nitrogen uptake capacity, and increase the number of nodules (Zhou et al., 2019; Zhang et al., 2022; Zheng et al., 2022). Research found that faba bean and maize intercropping significantly increased nodulation through root exudates from maize (Li et al., 2016). However, how the root exudates change before and after the soybean light environment changes in the maize-soybean strip relay intercropping system, and coexistence period, whether interaction with maize roots is beneficial for soybean nodulation is unknown.

In summary, the effect of crop root exudates on soybean nodulation ability is unclear under maize–soybean relay strip intercropping. We hypothesized that for maize–soybean strip relay intercropping, (a) increases in the enrichment of root flavonoids will result in a modified rhizobium structure; (b) the soybean nodulation and nitrogen fixation abilities will be strengthened during the different growth periods; and (c) maize root exudates will enhance soybean root growth and nodulation. Therefore, the maize–soybean relay strip intercropping system was used to conduct field experiments to analyze the flavonoid exudate components and contents in the roots of soybean varieties with different nodulation abilities. Combined with the pot experiment of simulating maize–soybean relay strip intercropping in the field, soybean root morphology and nodulation were observed after using maize root exudates to irrigate plants. The purpose of this study was to clarify the effects of environmental changes in the rhizosphere exudates of relay strip intercropping crops on soybean root growth, rhizobium diversity and nodule development.

## 2 Materials and methods

### 2.1 Experimental site

The field experiments were carried out in 2018 and 2019 at Renshou (30°16’N, 104°00’E), and the greenhouse experiments were conducted at Chongzhou. Both research sites belong to Sichuan Agricultural University, located in Sichuan Province, P.R. China. The climate data of the research location during the intercropping growth season from 2018 to 2019 are given in **Supplementary Figure S1**.

### 2.2 Experimental design and treatments

#### 2.2.1 Experiment 1

The maize variety used in this study was Denghai-605 (compact-maize), and the three genotypes of soybean varieties with distinct nodulation abilities were NTS1007 (NTS, supernodulation, provided by Queensland University, Professor Peter M·Gresshoff), Nandou-12 (ND, strongnodulation), and Guixia-3 (GX, weaknodulation). We used a modern maize–soybean relay strip intercropping system (**Supplementary Figure S2**), 2-rows of maize alternating with 2-rows of soybean (40cm-60cm-40cm-60cm), the maize hole was 17cm, 1 plant and soybean hole distance of 17cm, 2 plants. Monocropping soybean, a single soybean plant in a hole, was considered a control (**Supplementary Figure S2**). All varieties of soybean (NTS, ND and GX) and maize (Denghai-605) were maintained at the planting densities of 1.2×10^5^ plants ha^-1^ and 6×10^4^ plants ha^-1^, respectively. The field experiment was carried out using a randomized complete block design with 3 replications. The area of each replicate was 6×6 m. In addition, N, P and K were also applied as follows: 180 N kg/hm^2^ 、P_2_O_5_ 105 kg/hm^2^ and K_2_O 112.5kg/hm^2^ for maize, 60 N kg/hm^2^ 、P_2_O_5_ 63 kg/hm^2^ and K_2_O 52.5kg/hm^2^ for soybean, respectively. Maize was applied nitrogen fertilizer when sown (72 N kg/hm^2^) and 12-leaf stage topdressing, and soybean was applied nitrogen fertilizer at one time. The maize was sown in the first week of April and harvested in the last week of July, and the soybeans were sown in the second week of June and harvested in the first week of November in 2018 and 2019.

#### 2.2.2 Experiment 2

The soybean and maize cultivation substrate were mixtures of vermiculite and nutrient soil (V/V, 1:1). The soybean was inoculated with rhizobium complex bacterial agent (patent number: 201410442481.1; provided by Professor Youguo Li, Huazhong Agricultural University). A complete randomized block design was used consisting of two treatments (maize root exudate treatment, RE and distilled water treatment, DW), with 36 blocks.

A special trapezoidal device, made from metal, was used for planting (**Supplementary Figure S3**). The device was equipped with 3/4 volume of growing media. The middle of each cultivation area was planted with two maize plants (spacing of 15 cm), and the soybeans were planted on both sides (two plants on each side). A root exudate collector was set at the bottom of the maize planting position (**Supplementary Figure S3A**). The distance between maize and soybean was 40 cm. Each complete device grows four maize and eight soybean plants. The maize root exudates were collected as follows: rinsed with 5L distilled water until clean, stand for 6 hours. Then eluted with 1 L of distilled water. The eluant was used as root exudates for soybean treatment. Beginning from soybean seedling, the soybean was irrigated once every two days with 200 ml each time.

### 2.3 RNA extraction and quantitative real-time PCR analysis

Whole plants were excavated from experiments 1 and 2 at fifth trifoliolate (V5, vegetative growth period) and full bloom (R2, reproductive growth period) stage, roots were washed, and lateral roots were collected and stored in liquid nitrogen, three plants each replicate. The RNA from soybean roots without nodules was extracted using a polysaccharide polyphenol plant RNA extraction kit (DP441, TIANGEN, Beijing, China). Afterward, the total RNA was used for cDNA synthesis using a PrimeScript™ 1st Strand cDNA Synthesis Kit following the manufacturer’s instructions (TaKaRa, Japan). Quantitative real-time PCR (qRT–PCR) was performed on a QuantStudio 6 Flex system (Applied Biosystems, USA). In brief, a 20 µL volume of reaction solution contains 10 µL of TB Premix Ex Taq II (Takara, Japan), 2 µL of diluted cDNA, 1 µL of gene-specific primers (10 mM), and 6 µL ddH_2_O. The PCR amplification program was as follows: predenaturation at 95°C for 30 s, followed by 40 cycles of 95°C for 5 s, 55°C for 30 s and 72°C for 30s. The housekeeping gene Gm*Actin* was used as internal reference for soybean. Each sample test was set to produce three biological replicates. All of the primer sequences used in this study is listed in **Supplementary Table S1**.

### 2.4 DNA extraction and rhizobia sequencing

In experiment 1, at V5 stage, three soybean plants were excavated, and the roots were washed and collected nodules from the roots. DNA was extracted from root nodule samples, and PCR amplification with the *nifH* gene. Using the Illumina HiSeq 2500 sequencing platform, the raw data obtained was processed and filtered using internally written programs to perform the following treatments on the original sequencing data, resulting in effective tags. The diversity of rhizobia was compared and analyzed by QIIME software; UPARSE software was used to perform OTU clustering; The *nifH* database of Gaby and Buckley Lab (https://blogs.cornell.edu/buckley/nifh-sequencedatabase/) was used to annotate operational taxonomic units (OTUs), and Metastat was used to compare and analyze the different species of rhizobia.

### 2.5 Flavonoid extraction and HPLC analysis

Soybean root exudates were collected in experiment 1 at the V5 and R2 stages. The roots were rinsed with deionized water to remove various components adsorbed on the root surface and ensure the integrity of the root. Then, the soybean plants were placed in collection bags containing 500 ml of 0.0005 mol/L CaCl_2_ solution (Liu et al., 2019). Finally, the collection bags together with the plants were placed in the original growth environment for 2 h.

The soybean root exudate in the collection bags was extracted with ethyl acetate (Liu et al., 2019). An equal volume of ethyl acetate was extracted and repeated several times. Then, the extraction solution was evaporated to about 6ml under reduced pressure at 40°C on a rotary evaporator and adjusted to 10 ml with methanol. Finally, the samples were filtered through a 0.45 μm filter. The filtrate was analyzed for flavonoids using high-performance liquid chromatography (HPLC) using a Synergi 4u Hydro-RP 80A column (250 mm×4.6 mm, Phenomenex, USA) and detection at 270 nm (Liu et al., 2019). The elution was at 0.9 mL/min with solvents A (0.3% (v/v) acetic acid in ultra-pure water) and B (100% chromatographic pure methanol), with linear gradients from 30% to 40% B in 5min, from 40%—60% B in 5 min, from 60% to 90% B in 15 min, 90% B in 4 min, 90%—30% B in 5 min, 30% B in 4 min.

### 2.6 Nitrogenase activity

Nitrogenase activity within nodules in experiment 1 was measured by acetylene reduction assay (Chen et al., 2018).


$$\text{Nitrogenase activity }\left({\text{C}}_{2}{\text{H}}_{4}\text{ n mol}/\text{g}.\text{h}\right)={\text{n mol C}}_{2}{\text{H}}_{4}\;\text{produced}.\;{\text{g}}^{-1}\text{ fresh weight of nodule}.{\text{h}}^{-1}$$

$$\text{Nitrogen fixation potential}=\text{Nitrogenase activity}\times \text{nodule dry weight }\left(\text{g}.{\text{plant}}^{-1}\right)$$

### 2.7 Morphological analysis

The clean and intact roots at the V5 stage were scanned with an Epson Expression 10000XL scanner (Seiko Epson Co., Nagano-ken, Japan) and analyzed by WinRHIZO Pro5.0 software to obtain the following morphological traits: root length, root surface area and root volume. Root samples were cut and placed in 0.9% sodium chloride solution, and the number and length of root hairs within 2 cm from the root tip were observed with an optical microscope.

Six consecutive soybean plants were used to determine the nodule number, nodule dry weight, nodule size and root biomass in the 2018 and 2019 field experiments. In a pot experiment with irrigation maize root exudates treatment, the eight soybean plants were used to analyze the relationship between nodule growth, root biomass and maize root exudates. The whole soybean plants were excavated, and all nodules were meticulously collected from root and soil. After take picture, the nodules were enumerated and measured for size using Image Pro Plus 6.0 software. Nodules were classified by a diameter into large (≥2 mm) and small (<2 mm) groups (Peng et al., 2018). Plant root and nodule samples were oven-dried at 105°C for 30 min and 75°C until the weight was constant to obtain dry weight.

### 2.8 Statistical analysis

Statistical analysis of all data was conducted using the Graphpad Prism 9 software. Tukey’s multiple comparisons test and was performed to test the significant differences between intercropping and monocropping. The two-way or three-way ANOVA analysis was performed to determine if there were significant differences among treatment means and interactions. To compare the means at a 0.05, 0.01 and 0.001 significance level. The R language “vegan package” for Alpha diversity analysis; “tidyverse, ggsci, ggplot2 package” was used for data visualization and analysis; “cancor package” for Person correlation analysis.

## 3 Results

### 3.1 Root nodules and nodule size

**Figure 1:**
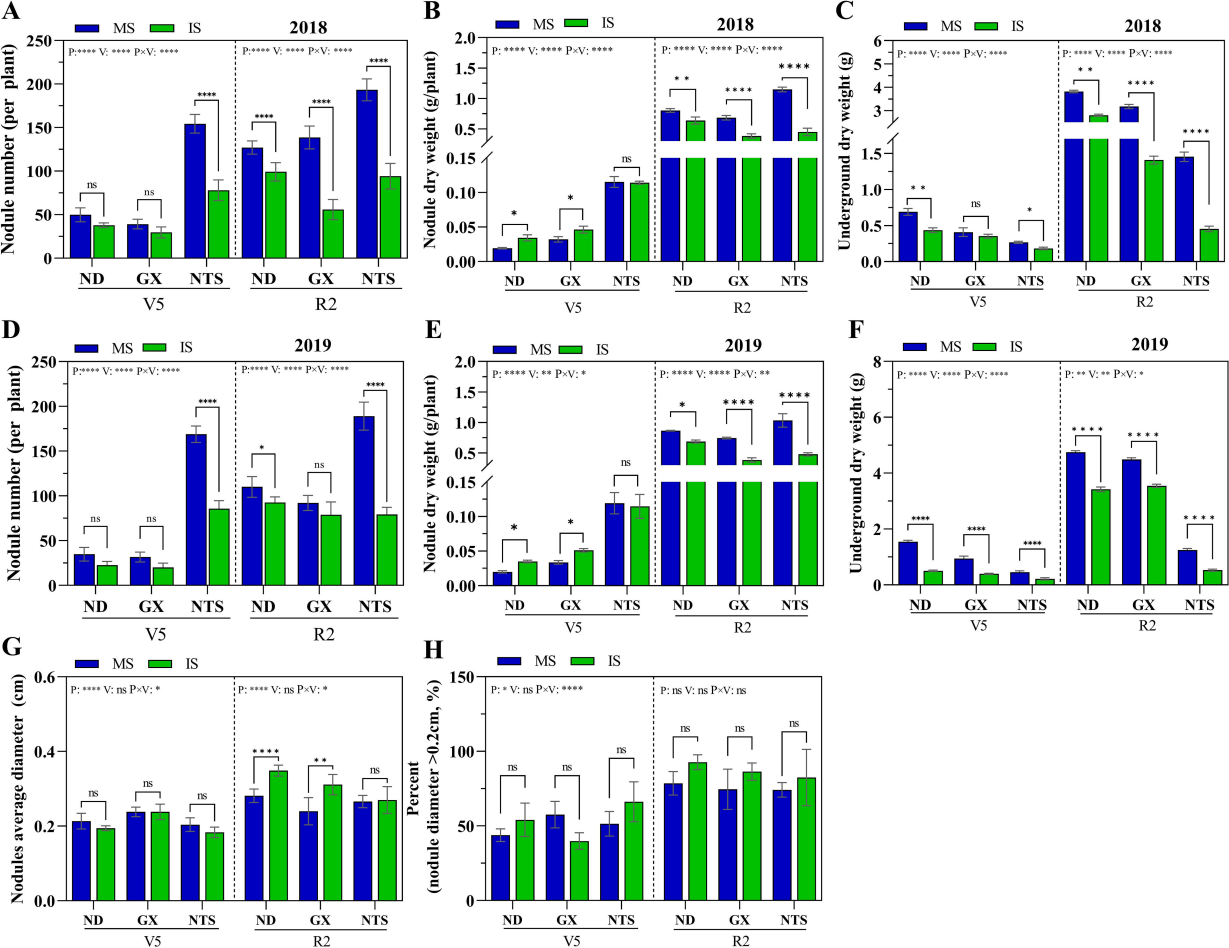
The nodule growth and development of soybean under different planting patterns. **(A–F)** The nodule number per plant, nodule dry weight and underground dry weight in 2018 and 2019; **(G)** Nodules average diameter; **(H)** Number of nodule with diameter>0.2cm as a percentage of total nodule number; Data were shown as mean ± S.E. (n=6 plants). P: Planting patterns; V: Varieties; P×V: the interaction between planting patterns and varieties; The asterisk “ns”, “*”, “**”and “****” indicate significant differences (P>0.05, P<0.05, P<0.01 and P<0.001) among treatments. MS, Monocropping soybean; IS, Intercropping soybean; V5, Five trifoliolate stage; R2, Flower full bloom stage.

At the V5 stage, the planting pattern and soybean variety interaction effect were significant effect on both nodule number and nodule dry weight (**Supplementary Table S2**). Under planting pattern, nodule number and nodule dry weight were significantly higher in NTS than ND and GX (**Figures 1A, B, D, E**). There was no significantly different nodule number in ND and GX in monocropping soybean and relay strip intercropping soybean, and its nodule dry weight was significantly higher in relay strip intercropping than monocropping, increasing by 78% and 43% in 2018, 76% and 52% in 2019 (**Figures 1B, E**). The nodule number of NTS in monocropping was higher than that of IS, more than 97% in two years (**Figures 1A, D**). At R2 stage, there was no significant difference in nodules number and nodule dry weight of ND and NTS between the two years. The nodules number of GX under monocropping increased significantly by 0.5-fold in 2018 compared with 2019. The nodules number of GX under relay strip intercropping was significantly 0.3-fold higher in 2019 than in 2018 (**Figures 1A, D**). The nodules dry weight of GX was not significantly different between 2018 and 2019. Compared with monocropped, the nodules number and nodule dry weight in intercropped soybean was reduced by 19% and 26% in ND, 37% and 85% in GX and 55% and 53% in NTS, respectively (**Figures 1A, B, D, E**). The nodule number and nodule dry weight of NTS under monocropping was the highest, and the highest in ND under relay strip intercropping.

The underground dry weight of soybean was significant three-factor interaction effect in different growth stages (**Supplementary Table S2**). At V5 stage, the underground dry weight under monocropping was significantly higher in 2019 than in 2018, and the ND, GX and NTS were significantly higher by 123%, 132% and 71%, respectively (**Figures 1C, F**). At R2 stage, the underground dry weight of ND and GX under monocropping were significantly higher by 24% and 41% in 2019 than in 2018. The underground dry weight of ND and GX under relay strip intercropping was 22% and 152% higher than that in 2018, respectively. Compared with monocropping, the underground dry weight of relay strip interctopping soybean significantly reduced, and the ND roots developed best among the varieties (**Figures 1C, F**). According to the analysis of nodule size, the percentage of nodule diameters greater than 0.2 cm was also higher than that in monocropping. At R2 stage, the nodule average diameter of ND and GX in relay strip intercropping was larger than that in monocropping, and the nodule average diameter of ND and GX significantly increased 26% and 21%, respectively (**Figures 1G, H**). Therefore, we further infer that the synergistic effect of crop root interaction and aboveground environment in maize–soybean relay strip intercropping leads to increase the nodule diameter.

**Figure 2:**
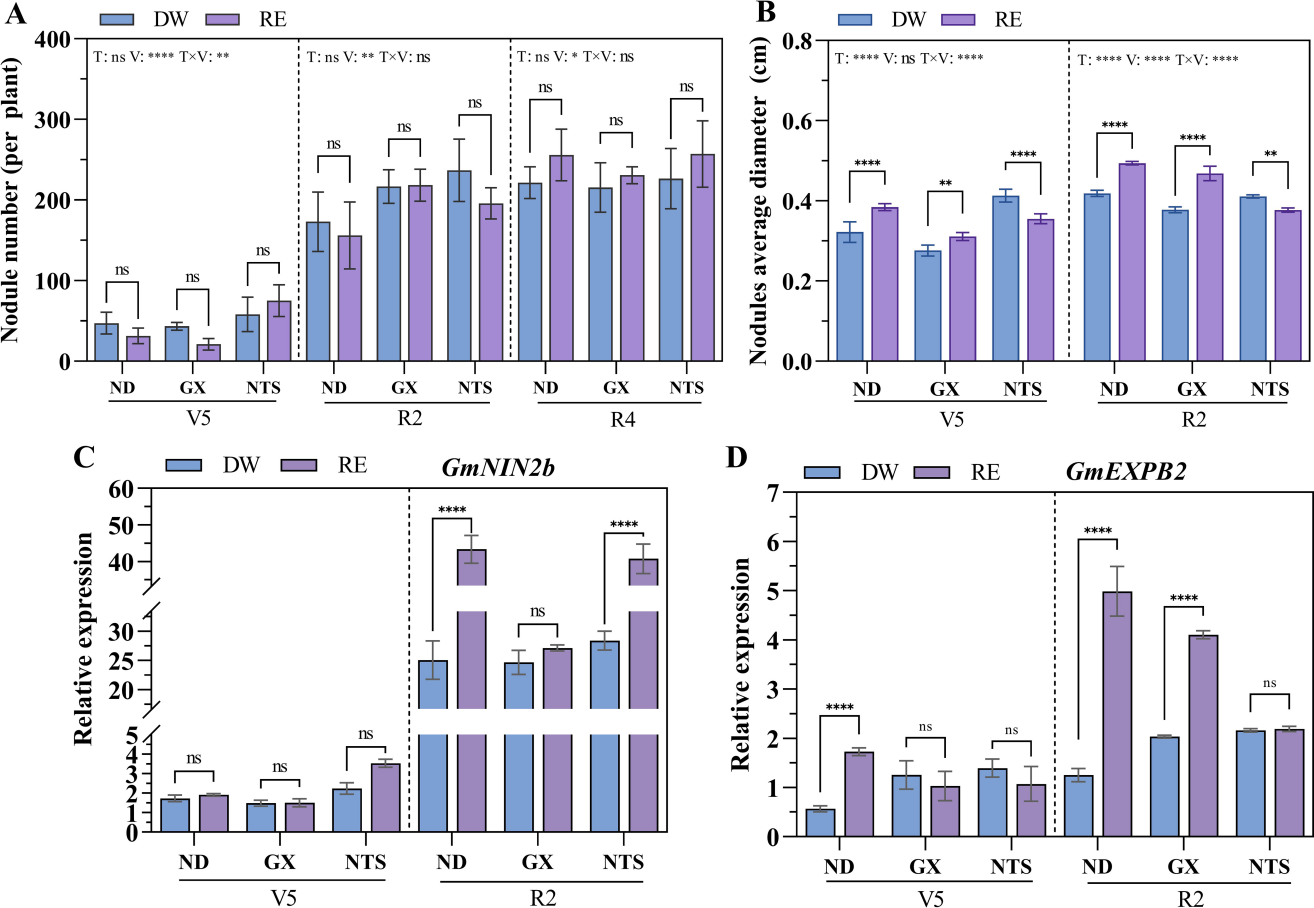
Effect of maize root exudates on the formation and development of nodules. **(A)** Nodule number per plant of soybean after treatment; **(B)** Nodule average diameter; **(C, D)** The expression of *GmNIN2b* and *GmEXPB2* in soybean after treatment. Data were shown as mean ± S.E. (n=8). T: Treatment; V: Varieties; T×V: the interaction between treatment and varieties; The asterisk “ns”, “*”, “**”and “****” indicate significant differences (P>0.05, P<0.05, P<0.01 and P<0.001) among treatments. V5: Five trifoliolate stage; R2: Full bloom stage; R4: Full pod stage; RE: Maize root exudates; DW, Distilled water.

At V5 stage, compared with the distilled water (DW) treatment, the root growth of ND and GX was promoted by maize root exudate (RE) treatment, while that of NTS was inhibited (**Supplementary Figures S4**, **S5A**). And after RE treatment, the nodule numbers of NTS increased significantly compared with ND and GX, while the dry weight of nodules was lower (**Figure 2A**, **Supplementary Figure S5B**). The nodule number of different soybean genotypes was significantly increased at R2 and R4 stages (**Figure 2A**). At R2 stage, the nodules number of GX and NTS under DW conditions, were significantly higher than ND by 23% and 37%, respectively, while under RE conditions, the nodules number of GX was significantly higher than ND by 40% (**Figure 2A**). At R4 stage, no significant difference in nodule number was observed under different treatments and varieties. The nodules dry weight of ND in RE treatment was higher than DW treatment at R2 and R4 stage (**Supplementary Figure S5B**). Compared with DW treatment, the nodule diameters of ND and GX with RE treatment were significantly higher and NTS significantly declined at V5 and R2 stages (**Figure 2B**). The nodule diameter was the largest in the NTS under DW treatment, while the ND was largest under RE treatment.

Next, we explored the effect of RE on soybean nodule formation-related genes. At V5 stage, The *GmNIN2b* expression level of NTS was higher than that of ND and GX, reflecting its super-nodulation characteristics. At R2 stage, *GmNIN2b* was up-regulated in the three genotypes of soybean, ND, GX and NTS with increases of 1.73-fold, 1.10-fold and 1.44-fold, respectively (**Figure 2C**). Additionally, the *GmEXPB2* expression of ND showed higher induced expression with RE treatment at V5 and R2 stages, increasing by 3.04-fold and 3.99-fold, respectively, which was conducive to root formation and nodule development. *GmEXPB2* expression in GX was significantly up-regulated by 1.02-fold at R2 stage after RE treatment (**Figure 2D**).

### 3.2 The flavonoid content of soybean roots in different planting patterns

**Figure 3:**
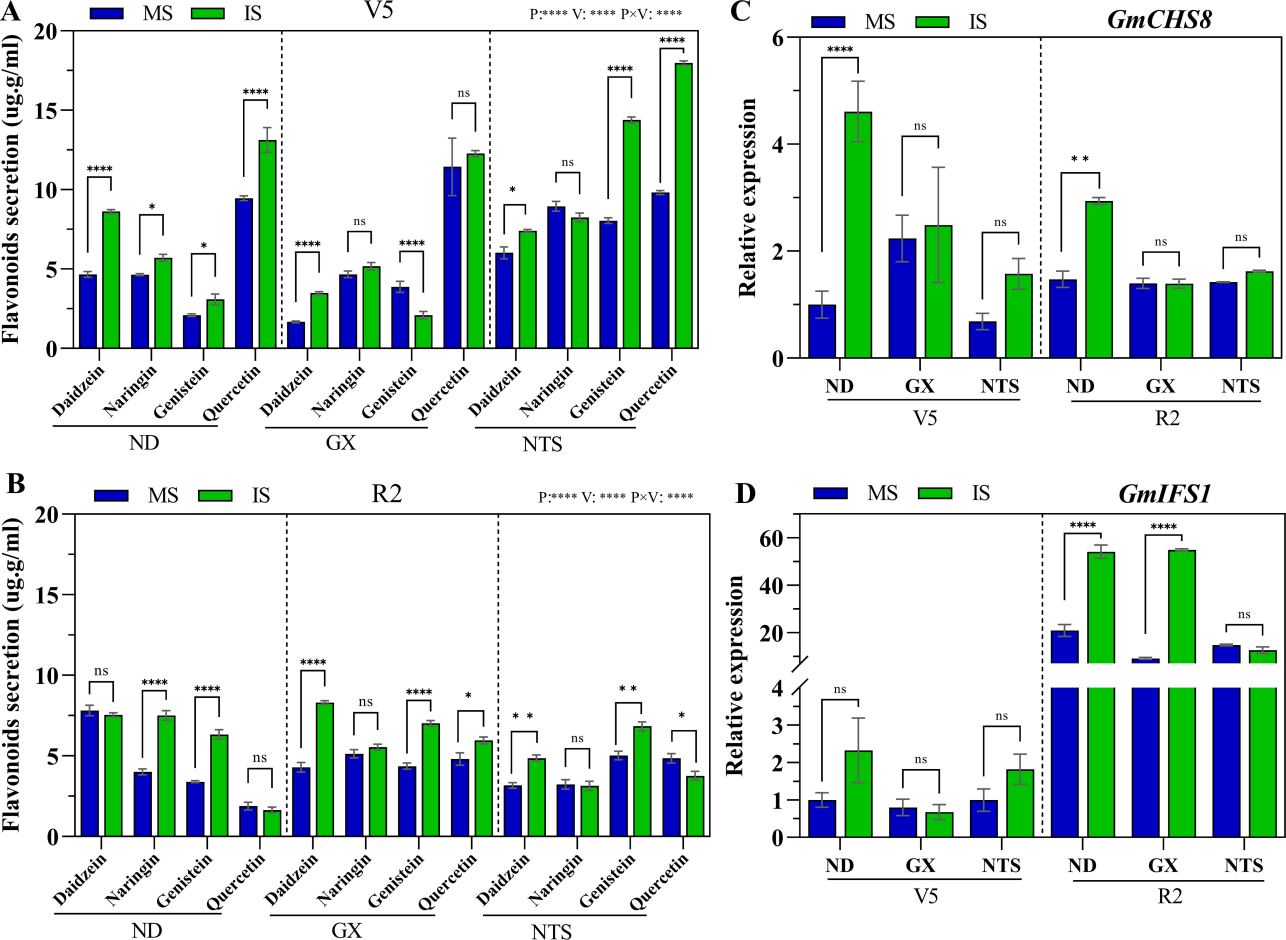
Flavonoids secretion **(A, B)** and expression of *GmCHS8* **(C)** and *GmIFS1* **(D)** genes by soybean roots at V5 and R2 under different planting modes. Data were shown as mean ± S.E. (n=4). The asterisk “ns”, “*”, “**”and “****” indicate significant differences (P>0.05, P<0.05, P<0.01 and P<0.001) among treatments. MS, Monocropping soybean; IS, Intercropping soybean; V5, Five trifoliolate stage; R2, Full bloom stage.

The flavonoid content of soybean root significantly differed in the two planting patterns and three genotypes (**Figure 3**). Compared with monocropping, at V5 stage, daidzein, naringin, genistein and quercetin contents of ND in relay strip intercropping were significantly increased by 85%, 23%, 48% and 39%, respectively (**Figure 3A**). The daidzein content of GX in relay strip intercropping were significantly greater by 109% and genistein observably down 46% than in monocropping at V5 stage (**Figure 3A**). The daidzein, genistein and quercetin contents of NTS in relay strip intercropping were evidently increased by 23%, 79% and 83% at V5 stage, respectively, compared with monocropping (**Figure 3A**). At R2 stage, the naringin and genistein contents of ND in relay strip intercropping were increased to a higher level of 87% and 86% than in monocropping, respectively. Compared with those of monocropping, under relay strip intercropping, the daidzein, genistein and quercetin contents were significantly enhanced by 94%, 61% and 24% in GX, and daidzein and genistein contents were increase up to 53% and 36% in NTS at R2 stage (**Figure 3B**).

The expression patterns of the key genes *GmCHS8* and *GmIFS1* for flavonoid synthesis in soybean roots were evaluated under different field cultivation patterns. The results showed that, compared with monocropping soybean, *GmCHS8* expression in the intercopping soybean was up-regulated at V5 stage. Specifically, ND and NTS were increased up to higher levels of 4.6-fold and 1.1-fold, respectively. However, the expression level of *GmIFS1* among all varieties was not significantly different at V5 stage under the two planting patterns (**Figures 3C, D**). In addition, *GmCHS8* and *GmIFS1* expression in ND was significantly up-regulated under relay strip intercropping at R2 stage, with levels reaching 2-fold and 2.4-fold higher than that under monocropping, respectively. The *GmIFS1* expression of GX was increased up to the highest level in relay strip intercropping compared with monocropping (**Figures 3C, D**).

### 3.3 Diversity of soybean rhizobia

**Figure 4:**
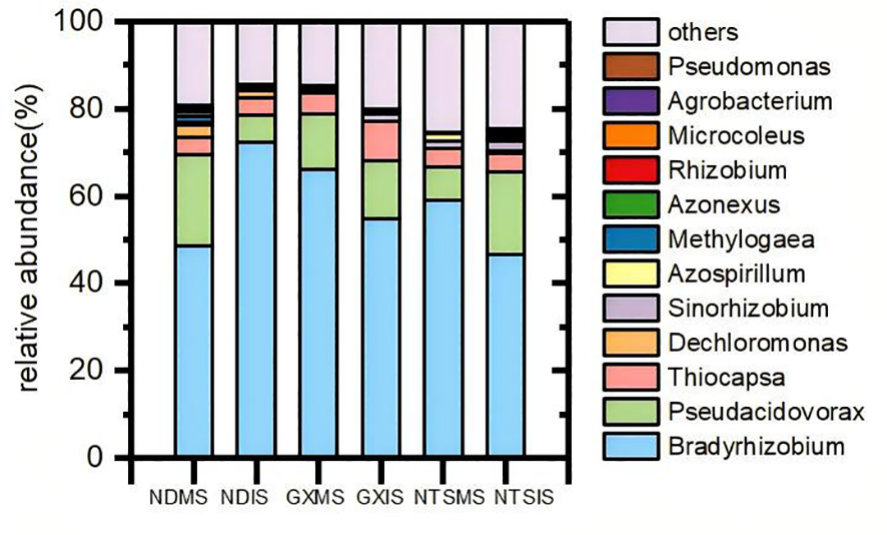
Community composition of soybean rhizobia under different planting patterns at V5 stage (2018). Colors represent the different nitrogen-fixing rhizobia; NDMS: Monocropping ND; NDIS: Intercropping ND; GXMS, GXIS, NTSMS and NTSIS same.

The species and relative abundance of nitrogen fixing rhizobium in different genotypes of soybean under monoculture and intercropping mainly included 12 genera, including *Bradyrhizobium* (58%), *Pseudacidovorax* (13%), and *Thiocapsa* (5%), accounted for a relatively high proportion (**Figure 4**). Then, the top 35 most abundant genera were compared by the Spearman correlation analysis (**Supplementary Figure S6**). In all samples, *Bradyrhizobium* and *Pseudovirorax* were closely and negatively correlated with each other, between *Dechromonas* and *Methylogaea* had a positive correlation, indicating that they may have antagonistic or synergistic effects on proliferation and function.

The α-diversity analysis (Shannon and Chao 1 indexes) showed that the Chao index showed a trend of ND>NTS>GX in monoculture and NTS>ND>GX in intercropping; the Shannon diversity index in monoculture showed the trend NTS>ND>GX, and in intercropping, it showed NTS>GX>ND (**Table 1**). The Shannon index value of ND and NTS were significantly different between MS and IS (**Table 1**). Intercropped ND was mainly manifested in the increase in *Bradyrhizobium* and *Sinorhizobium* and the decrease in other genera. The *Bradyrhizobium* is the main rhizobia genus of soybean nodule nitrogen fixation. Only the relative abundance of *Bradyzizobium* in intercropped ND was higher than that in monocropping, increasing by 47%. In contrast, intercropped GX and NTS exhibited a decline. The main reflection of intercropped NTS was enhanced in the number of *Methylogaea* and *Dechromonas*. In addition, the Chao index of GX was also significantly increased during intercropping, which was shown by the increase in other species of bacteria (**Figure 4**).

**Table 1:** Diversity indices.

Varieties	Planting patterns	Chao index	Shannon index
ND	MS	116.89 ± 10.84a	3.41 ± 0.17a
	IS	102.52 ± 8.07a	2.40 ± 0.16b
GX	MS	74.46 ± 9.52b	3.16 ± 0.12a
	IS	83.16 ± 10.88a	3.26 ± 0.14a
NTS	MS	113.38 ± 3.44a	3.43 ± 0.20b
	IS	119.11 ± 6.87a	4.18 ± 0.20a

MS, Monocropping soybean; IS, Intercropping soybean; The same variety significant differences were compared under different planting methods, and different letters in the same column indicated significant differences among samples (p<0.05).

### 3.4 Nitrogen-fixing capacity of soybean nodules

**Figure 5:**
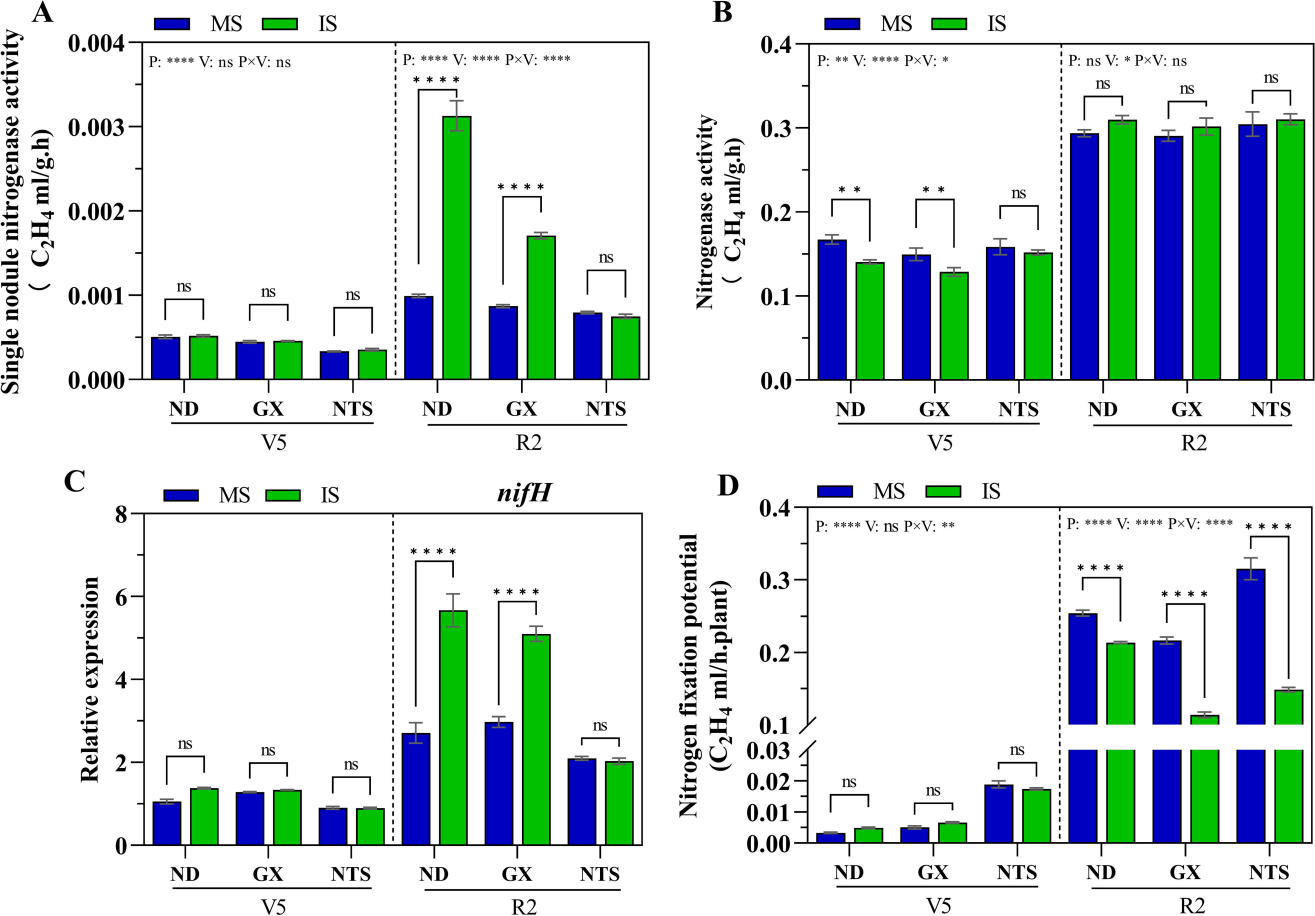
Nitrogen fixation capacity of soybean roots at V5 and R2 stage under different planting patterns. **(A)** Signal nodule nitrogenase activity; **(B)** Nitrogenase activity per plant; **(C)** The expression of *nifH* gene; **(D)** The nitrogen fixation potential of soybean. Data were shown as mean ± S.E. (n=6). P: Planting patterns; V: Varieties; P×V: the interaction between planting patterns and varieties; The asterisk “ns”, “*”, “**”and “****” indicate significant differences (P>0.05, P<0.05, P<0.01 and P<0.001) among treatments. MS, Monocropping soybean; IS, Intercropping soybean; V5, Five trifoliolate stage; R2, Full bloom stage.

The nitrogen-fixing capacity of nodules was further tested in 2019. At V5 stage, the nitrogen-fixing capacity of nodules was found to be low in three genotypes soybean (**Figure 5**). Compared with monocropping soybean, the nitrogenase activity of GX and ND was significantly decreased by 0.84-fold and 0.86-fold in the intercropping system (**Figure 5B**). At R2 stage, compared with monocropping, single nodule nitrogenase activity and the *nifH* gene in ND and GX was quickly increased during relay strip intercropping, and single nodule nitrogenase activity increased by 3.16-fold and 1.96-fold, respectively, the *nifH* gene was upregulated 2.71-fold and 1.82-fold, respectively (**Figures 5A, C**). Under relay strip intercropping condition, single nodule nitrogenase activity of ND was remarkably highest than GX and NTS. The nitrogenase activity in intercropping soybean was higher than that of monocropping soybean (**Figure 5B**). However, soybean’s nitrogen fixation potential in intercropping were significantly lower than that of monocropping, with decreased of 16% in ND, 47% in GX and 53% in NTS, respectively (**Figure 5D**). This indicated that a little number of nodules in monocropping soybean was reason for the decline soybean’s nitrogen fixation potential.

## 4 Discussion

### 4.1 Maize–soybean relay strip intercropping induced isoflavone exudates in soybean roots and improved nodule development

Gramineae/leguminous intercropping is widely considered as a sustainable agricultural production system that enhances nodulation and nitrogen fixation in legume plants, while as also promoting nitrogen uptake in populations (Neugschwandtner and Kaul, 2015; Xiao et al., 2018; Fan et al., 2019). Isoflavones, secreted by soybean roots, stimulate the expression level of rhizobium nodule genes, which are crucial for the formation of nodules and N_2_ fixation (Maj et al., 2010; Hassan and Mathesius, 2012). In this study, the secretion in soybean rhizosphere was significantly altered by maize–soybean relay strip intercropping. The daidzein and genistein contents of ND, GX and NTS significantly increased at V5 or R2 stage (**Figures 3A, B**). They are the most important isoflavones in the formation of soybean nodules (Peters et al., 1986; Smit et al., 1992). Under the coexistence of diverse crops, frequent chemical communication exists between the roots of interspecific plants, which cause specific sediment changes in the rhizosphere. However, compared with monocropping, the underground biomass and the number of root nodules per plant were both significantly decreased in three soybean genotypes under relay strip intercropping (**Figure 1**). The field root barrier experiments confirmed that under the interaction between maize and faba bean roots, the nodules dry weight increased in intercropping faba bean (Li et al., 2016). Meanwhile, in the maize-soybean relay strip intercropping system, soybean plants suffer from maize shading during their cogrowth period, and shaded leaves are thinner and smaller than those in normal sunlight, thus decreasing photosynthesis (Wu et al., 2017; Jiang et al., 2011). Several researches have also confirmed that stem diameter, root biomass, and plant biomass decrease under shading conditions (Yang et al., 2014, 2017). This indicates that soybeans exhibit a more robust secretion of isoflavones from the roots within in maize-soybean intercropping, facilitating enhanced the recruitment of rhizobia for nodulation. Additionally, shading also impacts the formation of root nodules.

Moreover, the expression level of isoflavone synthesis genes (*GmCHS* and *GmIFS*) increased at R2 stage in relay strip intercropping (**Figures 3C, D**). This upregulation facilitated the formation and development of root nodules. The synthesis of daidzein and genistein is first catalyzed by chalcone synthase (CHS) to synthesize an isoflavone skeleton. Subsequently, synthesized by an enzyme specifically present in leguminous plants-isoflavone synthase (IFS) (Sugiyama et al., 2016). Silencing of the key gene *GmIFS* revealed that two classes of soybean isoflavones, namely, daidzein and genistein, initiate nodulation (Subramanian et al., 2006).

The impacts of significant shading apply to all plants as excessive shade can trigger a carbon limitation. Being carbon limited, the plant will adjust (i.e., reduce) the amount of carbohydrates being transported to the roots (Liu et al., 2016; Fan et al., 2018). Therefore, all symbioses will suffer from the fact that the host plant is carbon-limited. According to previous research, shading inhibits the growth of underground roots and the occurrence of root nodules (Schwember et al., 2019). In this study, the nodules number of NTS was significantly higher than that in ND and GX. The growth and development of soybean nodules with different genotypes had different adaptability to stress. The genetic characteristics of soybean with strong nodulation still determined the occurrence of more lateral roots and nodule in the environment of maize soybean relay strip intercropping (Zhou et al., 2019). However, in this study, supernodulation soybean (NTS) was the lowest root biomass.

Soybean nodule became the “reservoir” of preferential supply of photosynthetic products after the removal of shading (Voisin et al., 2003), and the nodule weight increased rapidly. Meanwhile, due to the weak assimilation ability of the above-ground part, the rapid growth of the underground part and the accumulation of above-ground biomass were seriously affected (Yang et al., 2014). After maize harvest and the light recovery, the inhibition of soybean leaves functional traits by shading was alleviated (Fan et al., 2018; Wu et al., 2016), ND 12 and GX 3 showed more robust compensatory growth than monocropping (Chen et al., 2024). In this study, the root nodule numbers of ND and GX were higher than those in the shading stage (V5 stage), and the NTS showed no obvious change. At the same time, the nodule average diameter of ND and GX in relay strip intercropping was larger than that in monocropping, and there was no difference in NTS (**Figure 1**). The shade-tolerant variety ND12 still had high photosynthetic rate and leaf area index in the early shade environment, ensuring assimilation capacity (Chen et al., 2024). On the one hand, this confirms that the energy necessary for the growth and development of nodules is generated through plant photosynthesis (Schwember et al., 2019; Fujikake et al., 2003). On the other hand, it is speculated that maize-soybean relay strip intercropping is beneficial for nodule formation, with mainly focused on strong or weak nodulation soybeans, and super-nodulation varieties, such NTS, which appear to be less affected. Therefore, we consider that nodule number and energy support are the main factor resulting in lower nitrogenase activity during the co-growth period. After the end of the co-growth period, nodule size leads to differences in nitrogenase activity between monocropping and intercropping. The decrease in nitrogen fixation potential of intercropping soybean may be attributed to the number of nodules (**Figure 5**) (Tajima et al., 2015). This result also indicated that the lack of aboveground energy caused by the inhibition of the light environment was one of the factors that inhibited the formation of root nodules.

Shading inhibited the soybean growth, but relay intercropped soybean can be restored by the growth, to a certain extent to compensate for the growth disadvantage caused by the coexistence period (Wu et al., 2016). Indeed, strip intercropping can increase the harvest index and yield in grain production applications. Previously, we reported that the land-equivalent ratio of relay intercropped soybean grain yield was significantly elevated, realizing the high yield and high efficiency of relay intercropped soybean, and ND12 achieved the highest yield, which benefited from the compensatory growth of maize after harvest, and promoted the accumulation of nitrogen and the distribution of dry matter to grains (Chen et al., 2024).

### 4.2 Maize root exudates regulate soybean nodulation

As the growing site of soybean nodules, the growth status of roots directly affects the occurrence and formation of nodules (Schultze and Kondorosi, 1998). In this study, we found that maize root exudation was beneficial to soybean root growth (**Supplementary Figure S4**). The plasticity of soybean roots increased, which was more beneficial to the attachment of rhizobia. The rhizosphere exudates of cereal can promote legume nodulation (Li et al., 2016). Previous studies only investigated the response of legume root at seedling stage to maize root secretion, confirmed that in nonleguminous plants, neither barley nor wheat root exudates affected faba bean nodulation at seedling stage. Whereas maize enhanced nodulation through root exudates. The concentration of genistein in root exudates collected under the maize and faba bean co-culture was much higher than that of monocropping (Li et al., 2016). At the beginning of maize root exudates treatment (V5 stage), the nodule number and nodule dry weight of NTS was highest. However, at R2stage, GX was highest (**Figure 4**, **Supplementary Figure S5**). Meanwhile, Nodule diameters in ND and GX with maize root exudate treated were larger than distilled water treatment, while NTS declined (**Figure 2**). And the higher nodule numbers in soybean genotypes with maize root exudate treatment at the R4 stage. We considered that the deposition of maize root exudates in the soil continuously affects the growth of soybean roots and the secretion of nodulation compounds. However, due to the weak root growth and development of NTS, root interactive and communication with maize roots may be weak in maize soybean relay strip intecropping, resulting in less effect of maize root exudates on NTS nodulation.

Extracts from soybean have been reported to influence the production of lipochitooligosaccharides (Nod factor) in *Bradyrhizobium* stains (Lian et al., 2002), which show different attachment-specific surface properties when grown in soil-extracted solubilized organic matter (SESOM) from soybean fields (Sandhu et al., 2021, 2023). SESOM contains a large number of water diffusible compounds in the soil, the product of degradation compounds, plant exudates and absorbed compounds by microbiota (Liebeke et al., 2009; Sandhu et al., 2023). Therefore, the enrichment of root exudates of the two crops under maize-soybean relay strip intercropping may cause nodulation enhancement.

Additionally, the nodule size of GX and ND was boosted by maize root exudates (**Figure 2B**). It also enhanced the expression of genes involved in the early nodule process GmN1N2b in ND and the nodule development-related gene GmEXPB2 in ND and GX (**Figures 2C, D**), indicating that ND is more sensitive to maize root exudation. This is probably because the GmEXPB2 gene changed the root structure of soybean and promoted the development of root nodule primordium, resulting in increased nodule number and nodule mass (Li et al., 2015). NIN is one of the most important root nodule symbiotic genes as it is required for both infection and nodule organogenesis in legume (Fu et al., 2022). Taken together, our results suggest that maize root exudates are beneficial to soybean root growth, improving root area and increasing the possibility of rhizobia infection; increased rhizobia infection induces the expression of noodulation genes, such as NIN, and redulated developmental genes (GmEXPB2). The impact on sup-strong nodulation genotype soybeans is relatively small.

### 4.3 Rhizosphere exudates alter the structure of rhizobia and affect nodulation

The variation, diversity and abundance of the rhizobia community structure may be due to soil characteristics, the plant species grown in the soil, and the cultivation method applied (Yan et al., 2014). In this study, significant differences in the abundance of rhizobia communities were expressed between intercropping soybean and monocropping soybean (**Figure 4**). The difference was due to the interaction between different plant roots in intercropping. This interaction alters the root exudates of maize-soybean intercropping crops (Gao et al., 2014), and maize root exudates contain significant flavonoids and promote flavonoid synthesis in faba bean (Li et al., 2016). The evenness and complexity of nitrogen fixing microbial components increased (**Table 1**), which may be detrimental to the effectiveness of nodulation. Crops such as maize and rice selectively attract particular microorganisms via their secretions, which in turn foster plant growth and enhance nutrient uptake, thereby aiding their adaptation to new environments (He et al., 2022; Yu et al., 2021). In maize soybean intercropping, maize competition for nitrogen allows soybean to increase nitrogen fixation through favorable nodulation (Hu et al., 2017). Therefore, we considered that the reshaped rhizobium community structure and diversity improve the overall nitrogen fixation potential of soybean in the plant in the intercropping system.

## 5 Conclusion

Maize-soybean relay strip intercropping promotes root isoflavone exudates, such as daidzein and genistein, and changes the diversity of rhizobia in nodules. Nandou-12, showed higherrelative abundance of *Bradyrhizobium* in relay strip intercropping, while Guixia-3 and NTS1007 decreased. Meanwhile, nodulation of the NTS is most influenced by planting patterns. However, the growth and development of nodules was restored after recovery lighting, and mainly concentrated in Nandou-12 and Guixia-3. Irrigation with maize root exudates can promote nodule development in Nandou-12 and Guixia-3. And strengthen nodulation mainly in the late growth stage. Therefore, a favorable rhizosphere exudate environment can stimulate nodule development and improve nodule quality by promoting isoflavone exudates and then enhance nitrogen fixation efficiency.

## Data Availability

The original contributions presented in the study are included in the article/**Supplementary Material**. Further inquiries can be directed to the corresponding author.
