# Supplementary material for: Relay intercropped soybean promotes nodules development and nitrogen fixation by root exudates deposition
Source: Front Plant Sci. 2024 Dec 20;15:1447447. doi: 10.3389/fpls.2024.1447447 (PMC11695314; doi:10.3389/fpls.2024.1447447)
Supplement: Supplementary file 1 [file DataSheet1.docx]

Table S1 The specific sequences of the primer

| Primer Name | Sequence (5′-3′) |
| --- | --- |
| GmIFS1-RT-F | GACGCCTCACTTACGACAACTCTG |
| GmIFS1-RT-R | CCTGAGCTTGTTGACGGTGGTG |
| GmCHS8-RT-F | CCAGCAACCATCCTTGCCAT |
| GmCHS8-RT-R | TTGTCACACATGCGCTGGAA |
| GmActin-RT-F | GGCTGGTTTTGCTGGAGAT |
| GmActin-RT--R | ATCCTTCTGCCCCATCCC |
| GmNIN2b-RT-F | GCGATGGCGATCTTGAGGAGTG |
| GmNIN2b-RT-R | GAAGGAGTGCTGGTGGTGTTGG |
| GmEXPB2-RT-F | CATGCTTCCGCCCAATGGTACTC |
| GmEXPB2-RT-R | AGAATGGTGCCACAAGTGCTGAC |
| NifH-F | TGYGAYCCNAARGCNGA |
| NifH-R | ADNGCCATCATYTCNCC |


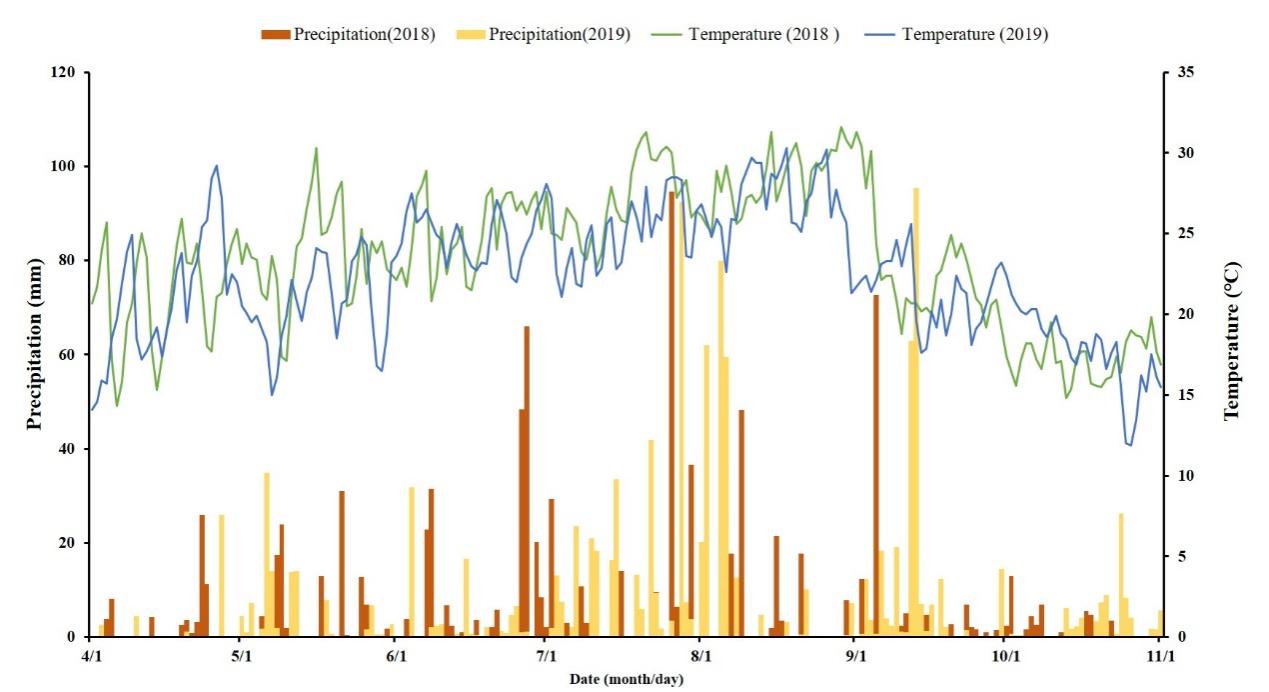


Fig S1 Daily temperature and precipitation during the crop growth season in 2018 and 2019


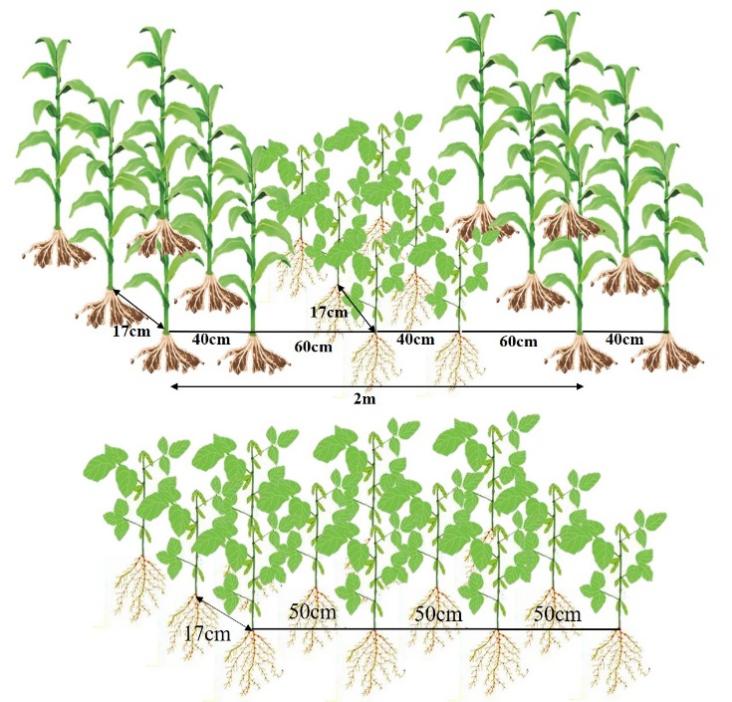


Fig S2 Schematic diagram of maize-soybean relay intercropping and monocropping soybean


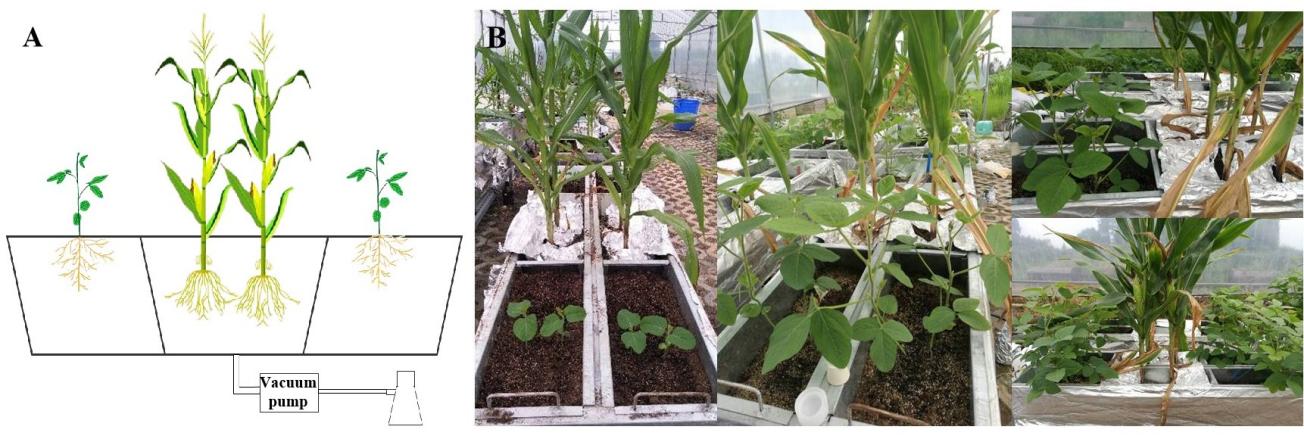


Fig S3 The diagram of maize root exudate collector. A: Model diagram; B: Indoor planting and collection.

Table S2 Variance analysis of soybean nodule and underground biomass under planting patterns and varieties

|  | V5 | | | R2 | | |
| --- | --- | --- | --- | --- | --- | --- |
|  | NN | NDW | UDW | NN | NDW | UDW |
| Y | 5.45 * | 1.32 ns | 496.51**** | 16.415**** | 90.06**** | 2264.97**** |
| P | 364.95**** | 27.84**** | 821.82**** | 457.866**** | 92.13**** | 4410.73**** |
| V | 1096.71**** | 966.00**** | 523.52**** | 105.180**** | 33.14**** | 9956.47**** |
| Y×P | 0.71 ns | 0 ns | 341.42**** | 17.933**** | 730.21**** | 59.29**** |
| Y×V | 19.60**** | 0.19 ns | 60.71**** | 0.073ns | 3.17* | 918.93**** |
| P×V | 162.80**** | 11.70**** | 123.71**** | 78.361**** | 8.47**** | 72.93**** |
| Y×P×V | 0.28 ns | 0.32 ns | 49.25**** | 19.277**** | 21.97**** | 93.23**** |

Note: V5:the fifth trifoliolate stage; R2: the full bloom stage; NN: Nodule number; NDW: Nodule dry weight; UDW: Underground dry weight; Y: Year; P: Planting patterns; Y×P: the interaction between year and planting patterns; Y×V: the interaction between year and varieties; P×V: the interaction between planting patterns and varieties; Y×P×V: the interaction between year, planting patterns and varieties; The ns, *, ** and **** indicate significant differences (P>0.05, P<0.05, P<0.01 and P<0.001) among treatments.


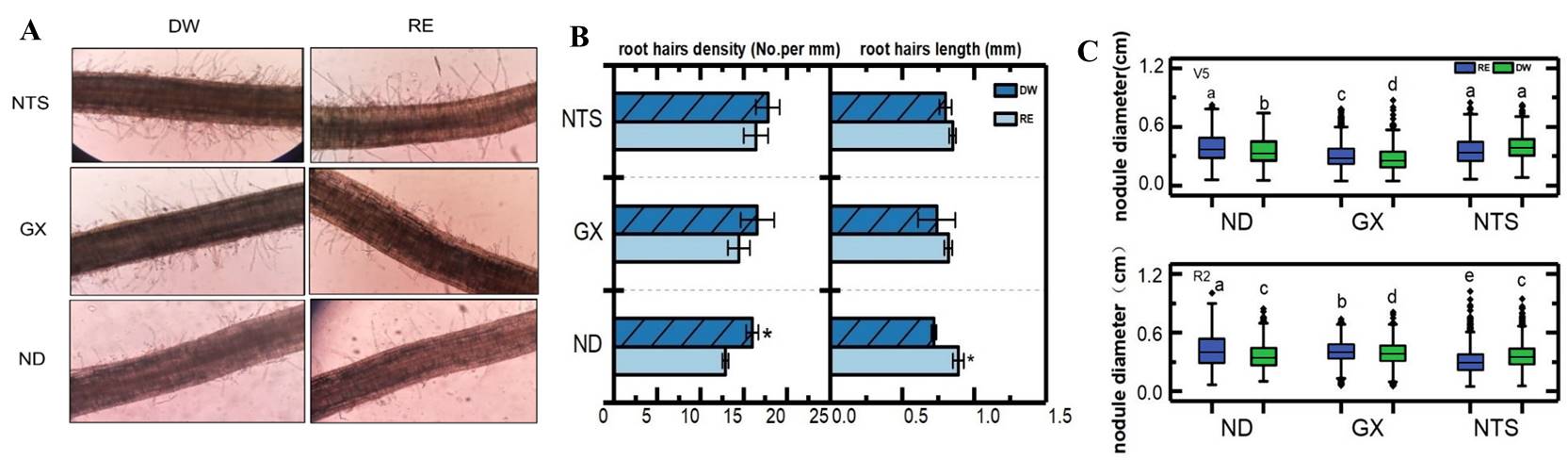

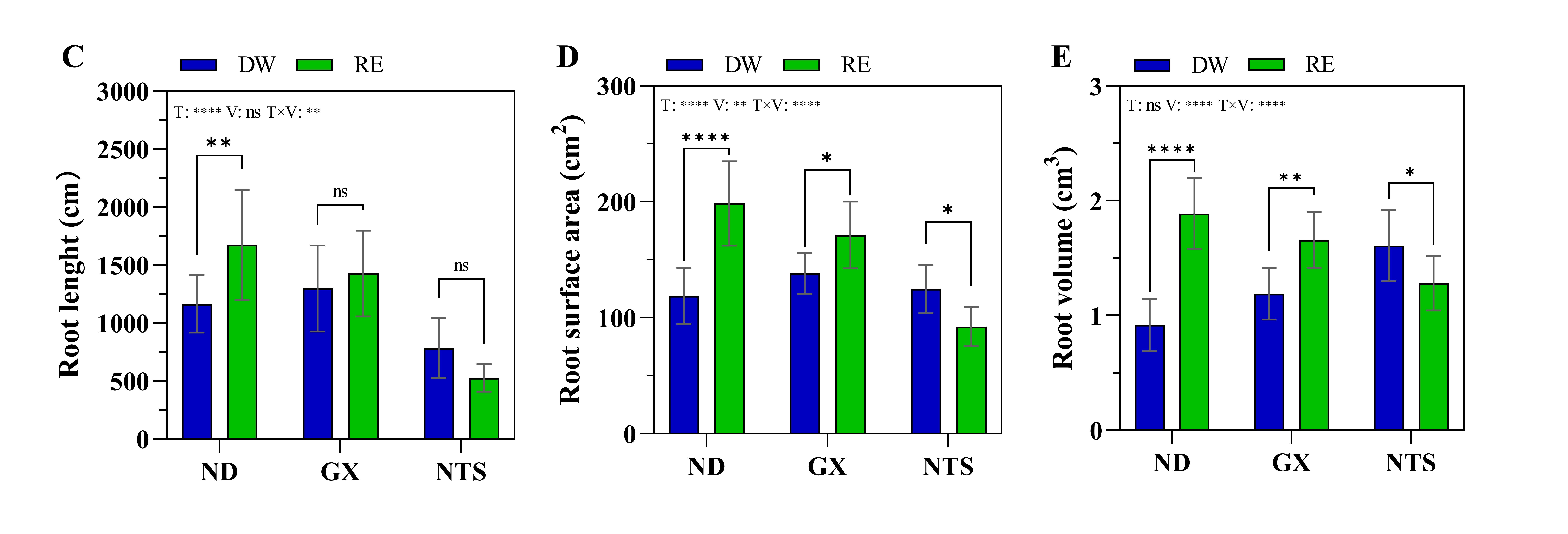


Fig S4 Root development of soybean after maize root exudation treatment at V5 stage. A-B: Root hairs; C: Root length, D: Root surface area, E: Root volume. Data were shown as mean ± S.E. (n=8). T: Treatment; V: Varieties; T×V: the interaction between treatment and varieties; The asterisk “ns”, “*”, “**”and “****” indicate significant differences (P>0.05, P<0.05, P<0.01 and P<0.001) among treatments. V5: Five trifoliolate; RE: Maize root exudates; DW: Distilled water.


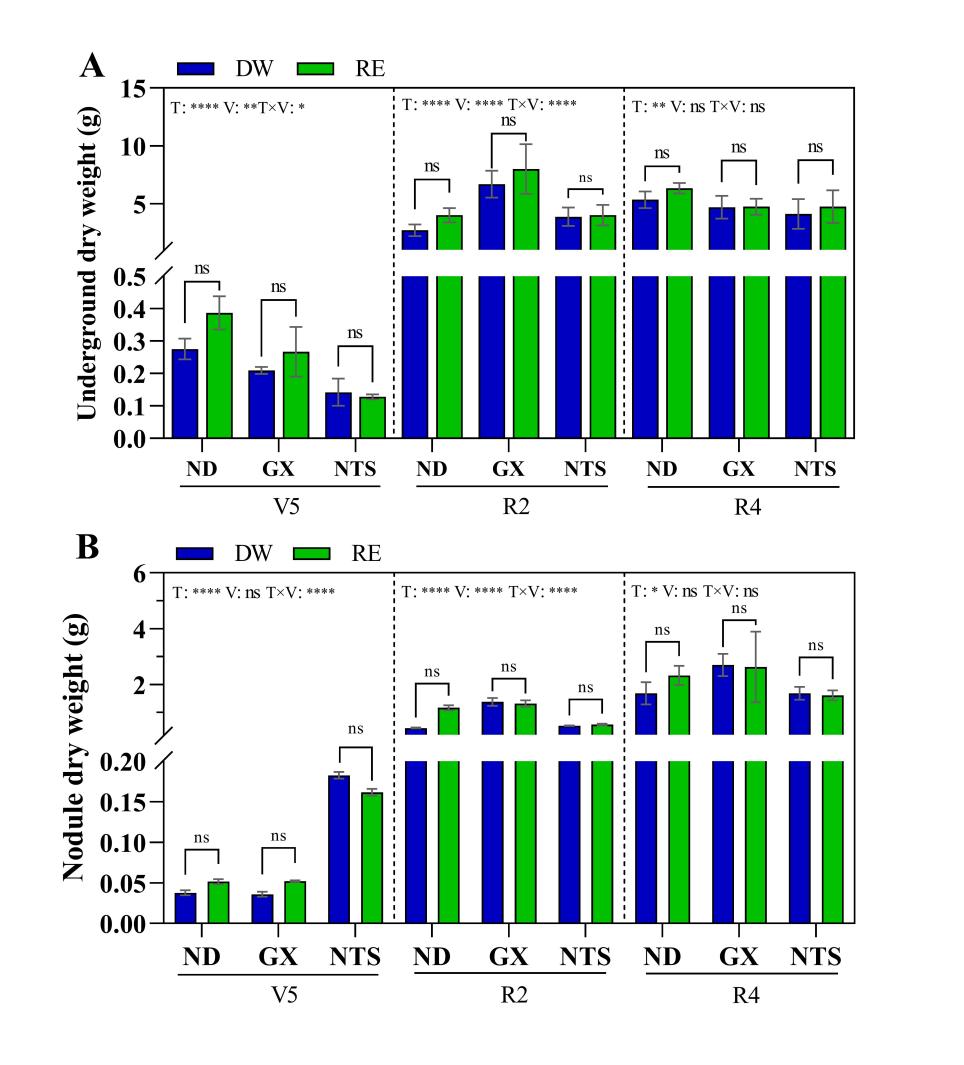


Fig S5 Underground biomass (A) and nodule dry weight (B) of soybean at different stages after treatment of maize root secretion. Data were shown as mean ± S.E. (n=8). T: Treatment; V: Varieties; T×V: the interaction between treatment and varieties; The asterisk “ns”, “*”, “**”and “****” indicate significant differences (P>0.05, P<0.05, P<0.01 and P<0.001) among treatments. V5: Five trifoliolate; R2: Full bloom; R4: Full pod; RE: Maize root exudates; DW: Distilled water.


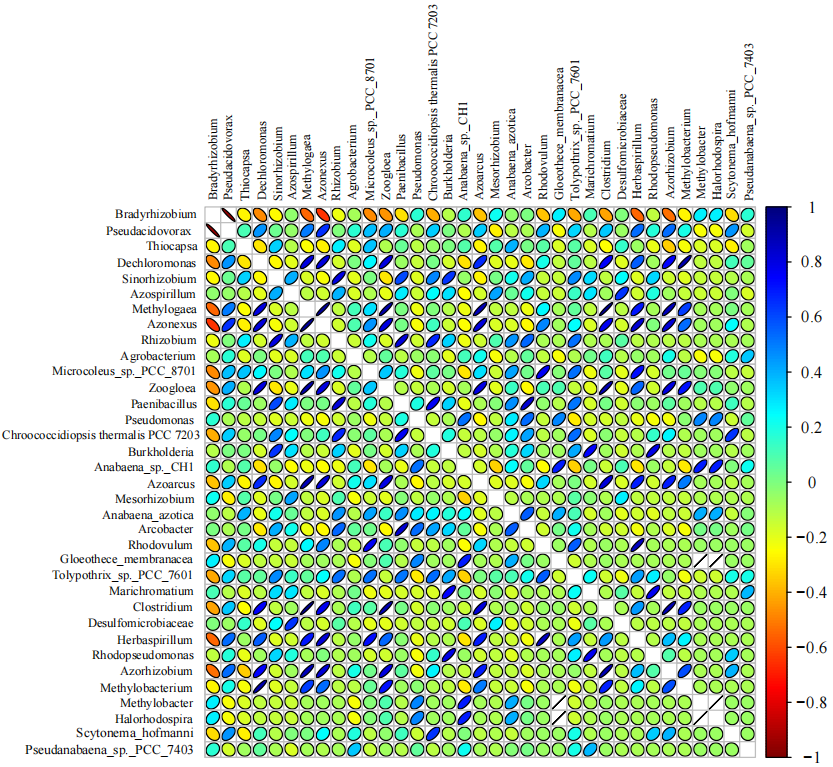


Fig S6 Species correlation of soybean rhizobia under different planting patterns
